# Evaluation of Acuros XB algorithm based on RTOG 0813 dosimetric criteria for SBRT lung treatment with RapidArc

**DOI:** 10.1120/jacmp.v15i1.4474

**Published:** 2014-01-06

**Authors:** Suresh Rana, Kevin Rogers, Shyam Pokharel, ChihYao Cheng

**Affiliations:** ^1^ Department of Medical Physics ProCure Proton Therapy Center Oklahoma City OK; ^2^ Department of Radiation Oncology Arizona Center for Cancer Peoria AZ; ^3^ Department of Medical Physics 21st Century Oncology Fort Myers FL; ^4^ Department of Radiation Oncology Vantage Oncology West Hills CA USA

**Keywords:** Acuros XB, anisotropic analytical algorithm, RTOG 0813, VMAT, RapidArc, lung cancer, heterogeneity correction

## Abstract

The Radiation Therapy Oncology Group (RTOG) 0813 protocol requires the use of dose calculation algorithms with tissue heterogeneity corrections to compute dose on stereotactic body radiation therapy (SBRT) non‐small cell lung cancer (NSCLC) plans. A new photon dose calculation algorithm called Acuros XB (AXB) has recently been implemented in the Eclipse treatment planning system (TPS). The main purpose of this study was to compare the dosimetric results of AXB with that of anisotropic analytical algorithm (AAA) for RTOG 0813 parameters. Additionally, phantom study was done to evaluate the dose prediction accuracy of AXB and AAA beyond low‐density medium of different thicknesses by comparing the calculated results with the measurements. For the RTOG dosimetric study, 14 clinically approved SBRT NSCLC cases were included. The planning target volume (PTV) ranged from 3.2‐43.0 cc. RapidArc treatment plans were generated in the Eclipse TPS following RTOG 0813 dosimetric criteria, and treatment plans were calculated using AAA with heterogeneity correction (AAA plans). All the AAA plans were then recalculated using AXB with heterogeneity correction (AXB plans) for identical beam parameters and same number of monitor units. The AAA and AXB plans were compared for following RTOG 0813 parameters: ratio of prescription isodose volume to PTV (R100%), ratio of 50% prescription isodose volume to PTV (R50%), maximal dose 2 cm from the PTV in any direction as a percentage of prescription dose $\left(D_{2\text{cm}}\right)$, and the percentage of ipsilateral lung receiving dose equal to or larger than 20 Gy $\left(V_{20}\right)$. The phantom study showed that the results of AXB had better agreement with the measurements, and the difference ranged from −1.7% to 2.8%. The AAA results showed larger disagreement with the measurements, with differences from 4.1% to 12.5% for field size $5\times 5\;{\text{cm}}^{2}$ and from 1.4% to 6.8% for field size $10\times 10\;{\text{cm}}^{2}$. The results from the RTOG SBRT lung cases showed that, on average, the AXB plans produced lower values for R100%, R50%, and $D_{2\text{cm}}$ by 4.96%, 1.15%, and 1.60%, respectively, but higher $V_{20}$ of ipsilateral lung by 1.09% when compared with AAA plans. In the set of AAA plans, minor deviation was seen for R100% (six cases), R50% (nine cases), $D_{2\text{cm}}$ (four cases), and $V_{20}$ (one case). Similarly, the AXB plans also showed minor deviation for R100% (one case), R50% (eight cases), $D_{2\text{cm}}$ (three cases), and $V_{20}$ (one case). The dosimetric results presented in the current study show that both the AXB and AAA can meet the RTOG 0813 dosimetric criteria.

PACS number: 87.55.D‐, 87.55.dk, 87.55.kd, 87.55.km

## I. INTRODUCTION

According to the most recent statistics released by the American Cancer Society, lung cancer will be the second most commonly diagnosed cancer in 2013, with an estimation of 118,080 and 110,110 new cases in men and women, respectively.^(1)^ Furthermore, lung cancer is the leading cancer killer in both men and women in the United States, with estimated deaths of 87,620 and 72,220 in men and women, respectively, in 2013.^(1)^ Currently, stereotactic body radiotherapy (SBRT) is used for the management of early stage non‐small cell lung cancer (NSCLC) as an alternative option to surgery.^(2)^ SBRT is a highly conformal technique that delivers high radiation dose with few treatment fractions to the tumor, while limiting the doses received by the critical structures.^(2)^

Several studies have reported that use of volumetric‐modulated arc therapy (VMAT) to treat SBRT lung cases reduces the treatment time, thus making the SBRT delivery using VMAT faster and more patient‐friendly compared to intensity‐modulated radiation therapy (IMRT).^3^, ^4^, ^5^ Despite the improvement in treatment efficiency, advanced imaging systems in the treatment rooms for accurate patient positioning, and high probability of tumor control (local control rates at three years up to 90%^(6)^) using SBRT, it faces the challenge in delivering the accurate dose to the tumor when inhomogeneous media are encountered along the photon beam path. The SBRT lung cases involve small fields with the presence of air, which causes the electronic disequilibrium effect near the air/tissue interfaces as the lateral range of secondary electrons becomes longer than the width of the small field segments.^(7)^ Thus, SBRT technique demands for accurate dose calculation algorithms that include tissue heterogeneity corrections; however, dose calculation algorithms employed in different treatment planning systems (TPSs) are not consistent in predicting doses, especially when heterogeneous media are involved.^7^, ^8^, ^9^, ^10^

At Arizona Center for Cancer Care, we currently use the Eclipse TPS (Varian Medical Systems, Palo Alto, CA) and anisotropic analytical algorithm (AAA), a convolution/superposition dose calculation algorithm which accounts for tissue heterogeneity corrections, for external‐beam radiation therapy treatment planning. The on‐going Radiation Therapy Oncology Group (RTOG) 0813 protocol requires the use of dose calculation algorithms with tissue heterogeneity corrections for the dose calculations on SBRT lung plans.^11^, ^12^ The current ROTG 0813 dose compliance criteria were established based on dose calculations computed by superposition algorithm.^11^, ^12^ Li et al.^(12)^ compared the SBRT plans calculated by Monte Carlo (MC) algorithm in Monaco TPS (Computerized Medical System, St. Louis, MO) with that of superposition algorithm in XiO TPS (Computerized Medical System) for SBRT NSCLC cases. That study reported RTOG 0813 dosimetric quantities of the MC calculations have larger magnitudes than those of the superposition calculations.^(12)^

Currently, Acuros XB (AXB), a new photon dose calculation algorithm similar to classic MC methods,^(13)^ is available in the Eclipse TPS. A number of studies have reported the better dose prediction accuracy of the AXB over AAA in the inhomogeneous media when compared with the measurements and MC simulations.^13^, ^14^, ^15^, ^16^, ^17^, ^18^ If the AXB is considered to be more accurate than the AAA for dose calculations, it is essential to investigate whether AXB can meet the RTOG 0813 dose compliance criteria. In the study by Li et al.,^(12)^ the results showed that RTOG 0813 dosimetric criteria may need adjustment when MC dose calculation algorithm is used. Recently, two studies^19^, ^20^ investigated the dosimetric impact of AXB and AAA for the SBRT lung cases; however, to our knowledge, no study has been published comparing the AXB and AAA calculations for the RTOG 0813 parameters on the computed tomography (CT) dataset of real patients with focus on RapidArc (Varian Medical Systems). The main purpose of this study was to compare the dosimetric results of AXB with that of AAA for RTOG 0813 parameters using real SBRT lung cancer treatment plans. Additionally, we have also evaluated the dose prediction accuracy of AXB and AAA beyond low‐density medium of different thicknesses by comparing the calculated results of AXB and AAA with the measurements.

## II. MATERIALS AND METHODS

### A. Dose calculation algorithms

Both the AAA and AXB are implemented in the Eclipse TPS (version 10.0.28). The AAA is an analytical photon dose calculation algorithm based on a pencil beam convolution/superposition technique.^(21)^ The tissue heterogeneity in the AAA is handled by scaling of primary photons and photon scatter kernel scaling in lateral directions according to local electron density.^22^, ^23^ In contrast, the AXB is considered to be similar to classic MC methods for accurate modeling of dose deposition in heterogeneous media.^(13)^ The AXB utilizes the linear Boltzmann transport equation (LBTE) and solves numerically that describes the macroscopic behavior of radiation particles as they travel through and interact with the matter.^(13)^ For detailed descriptions on the AXB and AAA, readers are advised to refer to publications by Vassiliev et al.^(13)^ and Tillikainen et al.,^(21)^ respectively.

### B. Dosimetric evaluation of AXB in inhomogeneous phantoms

**Figure 1:**
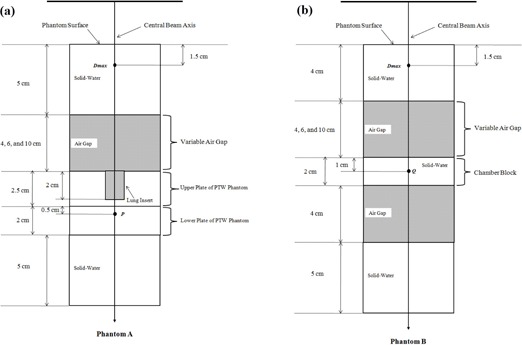
Schematic diagram of the experimental setup for the central axis depth dose computations and measurements in inhomogeneous phantoms: (a) Phantom A and (b) Phantom B The central axis percent depth doses (PDDs) at P and Q were calculated by normalizing the dose at P or Q to the dose at Dmax.

The dose prediction accuracy of AXB and AAA was evaluated in Phantom A and Phantom B (Fig. 1). The Phantom A was manufactured by combining solid water blocks and air gap of variable thickness with an acrylic PTW phantom (PTW‐Freiburg, Germany) for the purpose of taking measurements with a diode. Specifically, the Phantom A was composed of a 5 cm thick solid water as the top layer, which was followed by an air gap of variable thickness (4, 6, and 10 cm), an inhomogeneous PTW phantom, and a 5 cm thick solid water as the bottom layer, respectively. The inhomogeneous PTW phantom used in this study was composed of two plates: upper plate ($30\times 30\;{\text{cm}}^{2}$, 2.5 cm thick) with 2 cm deep central cavity of rectangular area $\left(15\times 15\;{\text{cm}}^{2}\right)$, and lower plate ($30\times 30\;{\text{cm}}^{2}$, 2.0 cm thick) (hereafter referred as diode block) with cavity for placement of PTW diode (Model: TN60017; nominal sensitive volume: 0.03 mm^3^). In the experimental setup of Phantom A, the central cavity of the upper plate was replaced by a 2 cm thick lung insert (15 cm long and 5 cm wide). The distance from the top surface of the diode block to the central cavity was 0.5 cm (i.e., diode's location was at the distance of 1 cm from the bottom surface of the lung insert, as shown in Fig. 1(a)).

The Phantom B was composed of a 4 cm thick solid water as the top layer, which was followed by an air gap of variable thickness (4, 6, and 10 cm), a 2 cm thick solid water (hereafter referred as chamber block), an air gap of 4 cm thickness, and a 5 cm thick solid water as the bottom layer, respectively. The Phantom B was manufactured to take the measurements with a cylindrical ionization chamber (Model: Exradin A1; Standard Imaging Inc., Middleton, WI; collecting volume: 0.053 cm^3^). In Phantom B, the chamber's location was at the distance of 1 cm from the top surface of the chamber block (i.e., 1 cm from the air gap/solid water interface, as shown in Fig. 1(b)).

#### B.1 Depth‐dose calculations

By mimicking the experimental set up as shown in Fig. 1, both the Phantoms A and B were scanned using General Electric (GE) LightSpeed CT scanner (GE Healthcare, Waukesha, WI) with 2.5 mm slices. The CT simulation was carried out with the PTW diode and ionization chamber placed inside diode block and chamber block, respectively. The digital imaging and communications in medicine (DICOM) CT datasets of scanned Phantoms A and B were then transferred to the Eclipse TPS. Central axis depth doses calculations in Phantoms A and B were computed by AAA (version 10.0.28) and AXB (version 10.0.28) using identical beam parameters (6 MV X‐ray from Varian Clinac iX accelerator, 100 cm SSD) for 200 monitor units (MUs). In Phantom A, we used a field size of $5\times 5\;{\text{cm}}^{2}$, whereas in Phantom B, we used field sizes of $5\times 5\;{\text{cm}}^{2}$ and $10\times 10\;{\text{cm}}^{2}$. For AXB, we chose dose‐to‐medium option to calculate the dose in the phantoms, and dose calculation grid size of 2.5 mm was used for both the AXB and AAA calculations.

#### B.2 Depth‐dose measurements

By keeping beam parameters identical to the ones that were used for dose calculations in the Eclipse TPS, 200 MUs were delivered to Phantoms A and B. In Phantom A, the measurement readings were acquired using PTW diode at depth of maximum dose (Dmax) and point P (Fig. 1(a)). In Phantom B, the measurement readings were acquired using a cylindrical ionization chamber at depth of Dmax and point Q (Fig. 1(b)). For both Phantoms A and B, the background readings were negligible compared to their true readings. At each point of interest (Dmax, P, and Q) of our experimental setup, measurement was repeated three times, and the average measured reading at P and Q was normalized to the average Dmax reading to obtain the percent depth dose (PDD). The measured PDDs were then compared with the PDDs computed by the AAA and AXB.

### C. Dosimetric evaluation of AXB for RTOG SBRT lung cases

#### C.1 CT simulation and contouring

Fourteen SBRT NSCLC cases previously treated with RapidArc at Arizona Center for Cancer Care were included in this retrospective study. During the CT scan of all 14 cases, patients were immobilized in a supine position on a GE LightSpeed CT scanner using wing board with an index bar, shoulder straps, and a knee roll. The CT images were acquired with 512 × 512 pixels at 0.25 cm slice spacing. The DICOM CT data were then electronically transferred to the Eclipse TPS for contouring and planning. The planning target volume (PTV) was created from a 5 mm wide isotropic expansion of the clinical target volume (CTV). The organs at risk (OARs), such as contralateral lung, ipsilateral lung excluding the PTV (ipsi‐lung), heart, and spinal cord, were delineated based on the axial CT images.

#### C.2 Planning and optimization

**Figure 2:**
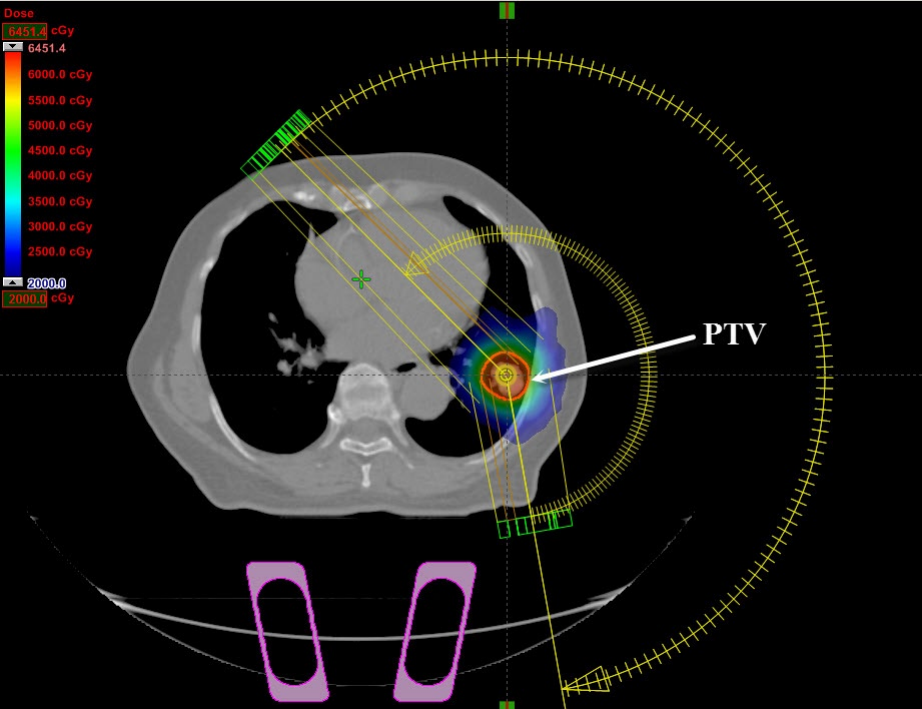
A transversal view of RapidArc plan setup in the Eclipse treatment planning system for SBRT NSCLC (case #6), showing the planning target volume (PTV).

Fourteen SBRT treatment plans for NSCLC cases were set up in the Eclipse TPS using RapidArc planning technique with two to four arcs (Fig. 2). For all the treatment plans, 6 MV X‐ray from Varian Clinac iX accelerator equipped with a Millennium 120 MLC (Varian Medical Systems) was used. The beam's eye view graphics in the Eclipse TPS were utilized to select the treatment plan's isocenter, which coincided with the center of the PTV. The field sizes of the coplanar arcs were chosen with the objective of achieving a maximal PTV coverage and a minimal OAR dose. All plans had a dose delivery schema of 60 Gy in 5 fractions, and plans were inversely optimized using progressive resolution optimizer (PRO), version 10.0.28, in order to meet the RTOG 0813 dosimetric criteria.

#### C.3 Dose calculation and normalization

The final dose calculations of the optimized plans were performed with the AAA with heterogeneity correction (version 10.0.28), and the resulting plans were referred as the AAA plans. The AAA plans were then generated based on the RTOG 0813 requirements^(11)^ as follows:

Each AAA plan was normalized such that 100% corresponds to isocenter of the plan.
2. The prescription isodose surface was selected such that it corresponds to 90% of the dose at the isocenter.
3. The prescription isodose surface chosen covered at least 95% of the target volume (PTV) and the 99% of the target volume (PTV) received a minimum of 90% of the prescription dose.

The AAA plans that had major deviation from the RTOG 0813 dosimetric criteria were reoptimized until these plans had either no deviation or minor deviation from the dosimetric compliance criteria. Next, for each case, the final AAA plan was recalculated with the AXB with heterogeneity correction (version 10.0.28) using dose‐to‐medium option for identical beam parameters (jaw settings, multileaf collimator positions, MUs, and number of arcs) as in the corresponding final AAA plan. A second set of treatment plans resulting from the AXB dose computation were referred as the AXB plans. The dose calculation grid size was set to 2.5 mm for all the AAA and AXB calculations in SBRT lung treatment plans.

#### C.4 Plan evaluation

The dose‐volume histograms (DVH) of all treatment plans (AAA and AXB) were generated in the Eclipse TPS. The AAA and AXB plans were then compared for the following RTOG 0813 dosimetric parameters:^(11)^

- R100%: ratio of prescription isodose volume to PTV,
- R50%: ratio of 50% prescription isodose volume to PTV,
- D_2cm_: maximal dose 2 cm from PTV in any direction as a percentage of prescription dose, and
- V_20_: percentage of ipsi‐lung receiving dose equal to or larger than 20 Gy.

In addition to RTOG 0813 parameters analysis, the average DVH of PTV was generated for the AAA and AXB plans by averaging the data over the 14 analyzed cases. For the purpose of comparison, the percent difference, D (%), in the RTOG 0813 parameters between the AXB and AAA plans was calculated using Eq. (1):

(1) $$D(x)=\left(\frac{\text{AXB}-\text{AAA}}{\text{AAA}}\right)\times 100$$

where *x* is the RTOG dosimetric parameter in the AXB and AAA plans.

## III. RESULTS

### A. Dosimetric evaluation of AXB in inhomogeneous phantom

Tables 1 and 2 show the difference between the measured and calculated (AAA and AXB) PDD data at point P in Phantom A (Fig. 1(a)) and at point Q in Phantom B (Fig. 1(b)), respectively. In Phantom A, the results of the AXB had better agreement with the measurements, and the difference between the AXB and measured PDD ranged from −0.1% to 2.8%, whereas the AAA results showed larger disagreement with the measurements, with differences from 6.5% to 8.5%. The results in Phantom B also demonstrated that the AXB was more accurate than the AAA. Specifically, the difference between the measured and AXB PDD ranged from 0.9% to 1.1% for field size $5\times 5\;{\text{cm}}^{2}$ and from −1.7% to 0.4% for field size $10\times 10\;{\text{cm}}^{2}$, whereas the difference between the measured and AAA PDD ranged from 4.1% to 12.5% for field size $5\times 5\;{\text{cm}}^{2}$ and from 1.4% to 6.8% for field size $10\times 10\;{\text{cm}}^{2}$.

**Table 1:** Comparison between the calculated (AAA and AXB) and measured percent depth dose (PDD) at point P in Phantom A (see Fig. 1(a)) for air gap thicknesses of 4, 6, and 10 cm. (6 MV photon beam, 100 cm SSD, 200 monitor units, $5\times 5\;{\text{cm}}^{2}$ open field size.)

	*DIODE*	*AAA*		*AXB*	
*Air Gap (cm)*	*PDD (%)*	*PDD (%)*	Δ^a^ (%)	*PDD (%)*	Δ^a^ (%)
4	65.2	69.5	6.5	65.1	−0.1
6	62.7	67.6	7.9	63.2	0.8
10	57.9	62.8	8.5	59.5	2.8

a ^a^ $\Delta (\text{PDD})=\times 100\frac{\left(\text{AAA or AXB}-\text{Diode}\right)}{\text{Diode}}$

AAA=anisotropic analytical algorithm; AXB=Acuros XB algorithm; PDD=percent depth dose.

**Table 2:** Comparison between the calculated (AAA and AXB) and measured percent depth dose (PDD) at point Q in Phantom B (see Fig. 1(b)) for air gap thicknesses of 4, 6, and 10 cm. (6 MV photon beam, 100 cm SSD, 200 monitor units, $5\times 5\;{\text{cm}}^{2}$ and $10\times 10\;{\text{cm}}^{2}$ open field sizes.)

*Field Size*: $5\times 5\;{\text{cm}}^{2}$					
	Chamber	*AAA*		*AXB*	
*Air Gap (cm)*	*PDD (%)*	*PDD (%)*	Δ^a^ (%)	*PDD (%)*	Δ^a^ (%)
4	74.5	77.5	4.1	75.2	0.9
6	69.4	74.2	6.9	70.1	1.1
10	61.7	69.4	12.5	62.4	1.0
*Field Size*: $10\times 10\;{\text{cm}}^{2}$					
	Chamber	*AAA*		*AXB*	
*Air Gap (cm)*	*PDD (%)*	*PDD (%)*	Δ^a^ (%)	*PDD (%)*	Δ^a^ (%)
4	78.4	79.5	1.4	77.1	−1.7
6	74.2	76.2	2.6	73.3	−1.3
10	66.8	71.4	6.8	67.1	0.4

a ^a^ A (PDD)

AAA=anisotropic analytical algorithm; AXB=Acuros XB algorithm; PDD=percent depth dose.

### B. Dosimetric evaluation of AXB for RTOG SBRT lung cases

**Figure 3:**
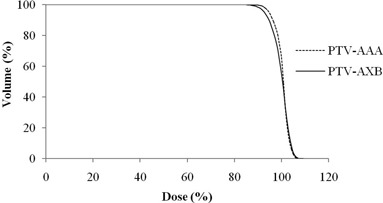
Cumulative dose‐volume histogram of planning target volume (PTV) in the AAA (dotted line) and AXB (solid line) plans averaged over the 14 analyzed SBRT lung plans generated with RapidArc technique. AAA=anisotropic analytic algorithm, AXB=Acuros XB algorithm, SBRT=stereotactic body radiation therapy.

Tables 3 to 6 show the results of RTOG 0813 dosimetric parameters for the 14 cases. The parameters that have minor deviations are marked in bold. Figure 3 shows the average DVH of the PTV (range, 3.2‐43.0 cc) for the AAA and AXB plans.

**Table 3:** Comparison of R100% in the AAA and AXB plans

		*R100%*			
*Case #*	*PTV Vol. (cc)*	*RTOG 0813 Minor Deviation*	*AAA Plans*	*AXB Plans*	*D (%)*
1	3.2	1.2‐1.5	1.13	1.09	−3.08
2	12.7	1.2‐1.5	1.35^a^	1.17	−13.19
3	13.1	1.2‐1.5	1.01	1.01	−0.38
4	16.0	1.2‐1.5	1.23^a^	1.10	−10.85
5	20.8	1.2‐1.5	1.29^a^	1.26^a^	−2.19
6	21.9	1.2‐1.5	1.13	1.09	−3.46
7	23.2	1.2‐1.5	1.13	1.05	−6.79
8	25.0	1.2‐1.5	1.14	1.12	−1.20
9	25.4	1.2‐1.5	1.09	1.04	−4.24
10	26.1	1.2‐1.5	1.21^a^	1.16	−4.32
11	27.5	1.2‐1.5	1.27^a^	1.17	−7.37
12	29.4	1.2‐1.5	1.23^a^	1.17	−5.10
13	30.7	1.2‐1.5	1.19	1.15	−3.28
14	43.0	1.2‐1.5	1.14	1.10	−3.99
AVG	22.71		1.18	1.12	−4.96
STDEV	9.57		0.09	0.07	3.56

a ^a^ Data that have minor deviations from RTOG 0813 criteria.

PTV=planning target volume; R100%=ratio of prescription isodose volume to PTV; AAA=anisotropic analytic algorithm; AXB=Acuros XB algorithm; D(%)=percent difference between R100%of AXB and AAA plans; AVG=average; STDEV=standard deviation.

**Table 4:** Comparison of R50% in the AAA and AXB plans

		*R50%*			
*Case #*	*PTV Vol. (cc)*	*RTOG 0813 Minor Deviation*	*AAA Plans*	*AXB Plans*	*D (%)*
1	3.2	5.63‐6.82	6.09^a^	6.10^a^	0.21
2	12.7	4.73‐5.82	5.59^a^	5.41^a^	−3.21
3	13.1	4.71‐5.80	5.18^a^	5.14^a^	−0.83
4	16.0	4.64‐5.70	4.84^a^	4.79^a^	−1.00
5	20.8	4.53‐5.54	5.01^a^	4.93^a^	−1.58
6	21.9	4.50‐5.50	4.50^a^	4.46	−0.87
7	23.2	4.48‐5.48	4.60^a^	4.53^a^	−1.47
8	25.0	4.45‐5.45	3.98	3.91	−1.57
9	25.4	4.44‐5.44	4.74^a^	4.72^a^	−0.32
10	26.1	4.43‐5.43	5.08^a^	5.03^a^	−1.00
11	27.5	4.41‐5.41	5.36^a^	5.34^a^	−0.28
12	29.4	4.38‐5.38	4.21	4.16	−1.11
13	30.7	4.36‐5.36	4.94^a^	4.89^a^	−1.19
14	43.0	4.13‐5.13	4.46^a^	4.37^a^	−1.93
AVG	22.71		4.90	4.84	−1.15
STDEV	9.57		0.56	0.56	0.83

a ^a^ Data that have minor deviations from RTOG 0813 criteria.

PTV=planning target volume; R50%=ratio of50%prescription isodose volume to PTV; AAA=anisotropic analytic algorithm; AXB=Acuros XB algorithm; D(%)=percent difference between R50%of AXB and AAA plans; AVG=average; STDEV=standard deviation.

**Table 5:** Comparison of $D_{2\text{cm}}$ in the AAA and AXB plans

		*D_2cm_(%)*			
*Case #*	*PTV Vol. (cc)*	*RTOG 0813 Minor Deviation*	*AAA Plans*	*AXB Plans*	*D (%)*
1	3.2	50.00‐57.00	35.40	35.90	1.41
2	12.7	50.00‐58.00	51.60^a^	51.40^a^	−0.39
3	13.1	50.00‐58.00	46.60	45.30	−2.79
4	16.0	51.27‐59.59	45.80	45.70	−0.22
5	20.8	53.45‐62.32	53.80^a^	52.40	−2.60
6	21.9	53.95‐62.94	45.90	45.20	−1.53
7	23.2	54.40‐63.50	51.70	51.10	−1.16
8	25.0	55.00‐64.25	46.40	45.20	−2.59
9	25.4	55.15‐64.43	49.60	49.00	−1.21
10	26.1	55.37‐64.71	54.50	54.10	−0.73
11	27.5	55.83‐65.29	57.20^a^	56.40^a^	−1.40
12	29.4	56.47‐66.08	47.90	46.50	−2.92
13	30.7	56.90‐66.63	61.30^a^	59.00^a^	−3.75
14	43.0	60.25‐73.06	53.60	52.22	−2.57
AVG	22.71		50.09	49.24	−1.60
STDEV	9.57		6.26	5.84	1.37

a ^a^ Data that have minor deviations from RTOG 0813 criteria.

PTV=planning target volume; $D_{2\text{cm}}=\text{maximal dose}2\text{cm from PTV in any direction as a percentage of prescription dose}$; AAA=anisotropic analytic algorithm; AXB=Acuros XB algorithm; $D(\%)=\text{percent difference between}\;D_{2\text{cm}}\;\text{of AXB and AAA plans}$; AVG=average; STDEV=standard deviation.

**Table 6:** Comparison of $V_{20}$ in the AAA and AXB plans

			*V_20_(%)*			
*Case #*	*PTV Vol. (cc)*	*Ipsi‐lung Vol. (cc)*	*RTOG 0813 Minor Deviation*	*AAA Plans*	*AXB Plans*	*D (%)*
1	3.2	1518.9	10‐15	2.03	2.06	1.48
2	12.7	2068.2	10‐15	3.21	3.12	−2.80
3	13.1	1226.2	10‐15	7.04	7.26	3.13
4	16.0	1909.7	10‐15	3.04	3.05	0.33
5	20.8	1505.7	10‐15	6.43	6.5	1.09
6	21.9	1432.7	10‐15	6.85	6.96	1.61
7	23.2	1917.5	10‐15	4.74	4.76	0.42
8	25.0	1124.2	10‐15	5.25	5.29	0.76
9	25.4	1515.4	10‐15	7.19	7.41	3.06
10	26.1	1112.4	10‐15	13.52^a^	13.54^a^	0.15
11	27.5	1312.0	10‐15	7.67	7.83	2.09
12	29.4	2071.3	10‐15	6.34	6.35	0.16
13	30.7	1282.9	10‐15	5.66	5.86	3.53
14	43.0	3199.3	10‐15	4.42	4.43	0.23
AVG	22.71	1656.9		5.96	6.03	1.09
STDEV	9.57	553.9		2.77	2.80	1.62

a ^a^Data that have minor deviations from RTOG 0813 criteria.

PTV=planning target volume; Ipsi−lung=ipsilateral lung; $V_{20}=\text{percentage of ipsilateral lung receiving dose equal to or larger than}20\;\text{Gy}$; AAA=anisotropic analytic algorithm; AXB=Acuros XB algorithm; $D(\%)=\text{percent difference between}\;V_{20}\;\text{of AXB and AAA plans}$; AVG=average; STDEV=standard deviation.

#### B.1 R100%

The R100% values were always lower in the AXB plans than in the AAA plans by an average difference of 4.96%±3.56 % (range, 0.38%−13.19%), and the results indicate that AXB plans demonstrated better high‐dose conformality. In the set of AAA plans, six cases showed minor deviation from the dose compliance criteria, whereas there was only one case with minor deviation in the set of AXB plans.

#### B.2 R50%

The R50% values were always lower in the AXB plans than in the AAA plans, except for case #1. The average difference in R50% was 1.15%±0.83% (range, 0.21%−3.21%), and the results indicate that AXB plans produced lower intermediate‐dose spillage. In contrast to R100% results, the number of cases that showed minor deviation from the dose compliance criteria increased for R50%. Specifically, 12 cases showed minor deviation for the AAA plans, and 11 cases showed minor deviation for the AXB plans.

#### B.3 $D_{2\text{cm}}$

The $D_{2\text{cm}}$ values were always lower in the AXB plans than in the AAA plans, except for case #1. The average difference in $D_{2\text{cm}}$ was 1.60%±1.37% (range, 0.22%−3.75%) with the AXB plans resulting in lower low‐dose spillage. In the set of AAA plans, four cases showed minor deviation from the dose compliance criteria, whereas three cases had minor deviation in the set of AXB plans.

#### B.4 *V_20_*

The $V_{20}$ values of ipsi‐lung were always higher in the AXB plans than in the AAA plans, except for case #2. On average, the $V_{20}$ values were higher in the AXB plans than in the AAA plans by 1.09%±1.62%. Both sets of plans (AAA and AXB) had one case showing minor deviation from the protocol criteria.

## IV. DISCUSSION

Photon dose calculations present challenges especially when a MV X‐ray beam travels through media of different density. The accurate modeling of primary beam attenuation and lateral scatter due to the presence of different media heterogeneities along the photon beam path is essential in order to avoid the dose overestimation or underestimation. Furthermore, when irradiation of a lung tissue is involved, dose calculation algorithms must have tissue heterogeneity corrections that will account accurately for the electron transport near air/tissue interface. The results from our phantom studies have shown an agreement with other studies, which reported that the AXB is more accurate for dose calculations in heterogeneous media compared to the AAA. Bush et al.^(14)^ showed that the AXB differences with MC results up to 4.5% and the difference between the AAA and MC results was up to 10.2% and 17.5% in lung and low‐density lung, respectively. Rana and Rogers^(16)^ showed the discrepancies between the AXB and measured data up to 3.8%, whereas the AAA differences with the measurement up to 10.9% beyond the air gap in a rectangular heterogeneous phantom. Similar observation was made by Kan et al.^(18)^ who reported that the AAA and AXB differed from the measurements by up to 10% and 3%, respectively, in the anthropomorphic phantom. Furthermore, in our phantom studies, we noticed that the discrepancy between the AAA and measured results increased with a larger air‐gap thickness for the same field size, but the discrepancy decreased with a larger field size for the same air‐gap thickness. Specifically, for a 10 cm air gap, the AAA showed a larger discrepancy (Phantom A: 8.5% for field size $5\times 5\;{\text{cm}}^{2}$; Phantom B: 12.5% for field size $5\times 5\;{\text{cm}}^{2}$ and 6.8% for field size $10\times 10\;{\text{cm}}^{2}$), whereas the AAA results for a 4 cm air gap showed smaller differences with the measurements (Phantom A: 6.5% for field size $5\times 5\;{\text{cm}}^{2}$; Phantom B: 4.1% for field size $5\times 5\;{\text{cm}}^{2}$ and 1.4% for field size $10\times 10\;{\text{cm}}^{2}$). For AXB, we observed an increasing difference (from −0.1% to 2.8%) between the calculated results and measurements with an increasing air‐gap thickness (from 4 cm to 10 cm) in Phantom A; however, no such discernible trend could be identified between the AXB and measured data in Phantom B for both the test field sizes. A larger air gap, such as the one used in this study, can cause the increased lateral scatter loss within the air gap, thus reducing scatter contribution to the points P and Q. Dose prediction errors shown by the AXB and AAA in this study may also be due to incorrect modeling of primary beam attenuation or lateral scatter contribution to the point beyond low‐density heterogeneity.

In the current study, the $V_{20}$ of ipsi‐lung was found to be higher in the AXB plans than in the AAA plans, and this result is consistent with the findings of Kathirvel et al.^(19)^ and Rana et al.^(20)^ If the AXB is considered to be more accurate than the AAA, our study shows that the AAA typically overestimates the magnitudes of R100%, R50%, and $D_{2\text{cm}}$, but underestimates the $V_{20}$ of the ipsilateral lung when compared to the AXB. The comparison between the AAA and AXB plans showed that the average difference in R100% is larger (D=4.96%) compared to ones in R50% (D=1.15%) and $D_{2\text{cm}}$ (D=1.60%). In general, we have observed that the PTV results of AXB were lower than that of AAA; however, for smaller PTV volumes, the AXB may not always produce lower values than that of AAA, especially for R50% and $D_{2\text{cm}}$. Specifically, for the smallest PTV volume (case #1) in this study, we noticed that the AXB predicted higher R50% and $D_{2\text{cm}}$. Furthermore, one must pay careful attention to the reduced PTV coverage from the AXB calculations compared to the AAA calculations, as shown in the Fig. 3. These dosimetric differences may be due to different beam modeling approach within the AAA and AXB in accounting electronic disequilibrium in different regions, such as in the high‐dose region near the target and in the intermediate or low‐dose regions away from the target.

In the study by Li et al.,^(12)^ it was reported that XiO superposition algorithm underestimated the RTOG 0813 results compared with the MC algorithm, which was shown to be more accurate than XiO. In contrast to the findings of Li and colleagues, our study showed that the AAA, a convolution/superposition algorithm,^(21)^ overestimated the magnitude of RTOG 0813 dosimetric parameters compared to the AXB, an algorithm similar to classic MC method.^(13)^ In addition to difference in dose calculation algorithms used in these two studies, Li et al.^(12)^ used the IMRT technique to generate the SBRT lung plans, whereas RapidArc technique was used for treatment planning in our study. The results between the Li study and our study showed that different dose calculation algorithms and treatment planning techniques may result into variation in the RTOG 0813 dosimetric results.

## V. CONCLUSIONS

In comparison to the AAA, the AXB is more accurate for dose prediction in the water‐equivalent material that is situated beyond the low‐density medium. The preliminary dosimetric results presented in the current study showed that both the AXB and AAA can meet the RTOG 0813 dosimetric criteria. On average, the AXB calculations produced lower magnitudes of R100%, R50%, and $D_{2\text{cm}}$, but higher $V_{20}$ of ipsi‐lung when compared to the AAA calculations.

## Supporting information

Supplementary Material
