# Supplementary figures and images for: Evaluation of Acuros XB algorithm based on RTOG 0813 dosimetric criteria for SBRT lung treatment with RapidArc
Source: J Appl Clin Med Phys. 2014 Jan 6;15(1):118–29. doi: 10.1120/jacmp.v15i1.4474 (PMC5711238; doi:10.1120/jacmp.v15i1.4474)

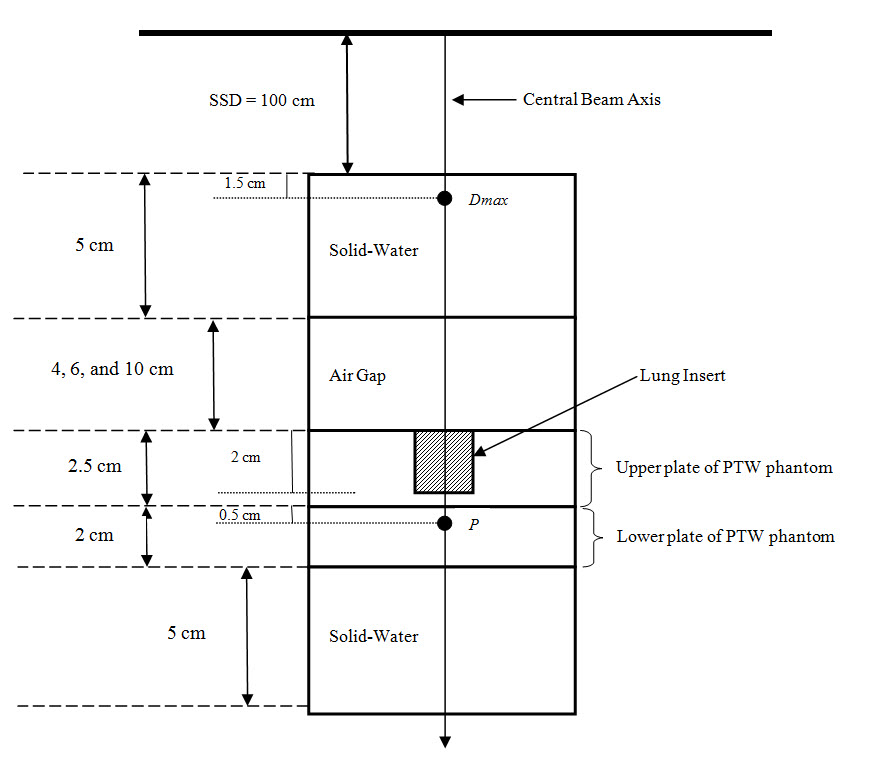

Supplement: Supplementary file 1 — Supplementary Material [file ACM2-15-118-s001.jpg]
